# Typhoidette

**Published:** 1902-04-12

**Authors:** 


### *TYPHOIDETTE.*

Among the many interesting matters dealt with by Professor Corfield in the Milroy Lectures we find reference to a question which urgently demands a solution, namely, the manner and extent to which persons habitually exposed to the infection of typhoid fever attain thereby immunity to the disease. It is easy to maintain that in a community constantly exposed to such infection a large proportion of the people are likely to have passed through attacks of the disease and thus to have become immune. But it may still further be suggested that they may have passed through attacks, not of true recognisable typhoid, but of a disease so modified as to be clinically as different from fully-developed enteric fever as varioloid is from small-pox, and may still have obtained thereby a considerable degree of immunity to the major malady. Dr. P. Lorain has said "Perhaps there is a *typhoidette* as there is a varioloid. There is no reason why one should not admit this hypothesis, but it is a hypothesis." Speaking of certain cases of so-called gastric disturbance, apparently not specific, but occurring alongside of other well-marked cases of typhoid in the course of an epidemic, Professor Brouardel says "That which Lorain considered as a hypothesis seems to me to-day absolutely proved, and I demand that one should admit in nosology this name of *typhoidette* which has the great advantage of recalling, more than the names actually used, the source from which some of these gastro-intestinal conditions originate." Professor Brouardel then asks whether these mild forms of infection afford a relative immunity from a fresh attack of the disease. "Does the typhoidette differ from typhoid fever as the varioloid does from variola?" He considers that it does so, and that to this cause we must attribute the relative immunity which the inhabitants of countries often visited by typhoid fever enjoy, and Professor Corfield adds, "this very fact has been observed among the Boers during the present war." The matter is one of much interest and importance, for this hypothesis suggests that while the sanitary improvements, on which all civilised countries are at the present time spending such vast sums of money, do undoubtedly diminish disease in the protected areas, they nevertheless may, perhaps, leave the people who are brought up among these improved conditions more liable to be attacked by fatal forms of filth diseases than those who from their birth onwards have been exposed to the various infections which are bred in dirt.

